# Oral Health, Immigrant Status, and Adult Children’s Support Among Chinese American Older Adults

**DOI:** 10.1093/geroni/igaa057.2902

**Published:** 2020-12-16

**Authors:** Nan Jiang, Bei Wu, Wei Zhang

**Affiliations:** 1 National University of Singapore, Singapore, New York, Singapore; 2 New York University, New York, New York, United States; 3 University of Hawaii at Manoa, Honolulu, Hawaii, United States

## Abstract

Adult children play an important role in older immigrants’ health outcomes. Research has indicated that older adults may benefit from adult children caregivers’ support for oral health. However, little is known about children’s support for improving oral health for older immigrants. Using the 2018 survey of 430 Chinese older adults age 55 and older in Honolulu, Hawai’i, we examine the associations among immigrant status, adult children’s support and perceived oral health for Chinese American older adults. Emotional support from adult children protects the self-rated oral health for the immigrant group, while financial support is linked to fewer oral health problems among the US-born group. Therefore, the current study underlines the importance of investigating different pathways among foreign-born and native-born Chinese older adults with regard to children’s support on their oral health outcomes. Part of a symposium sponsored by the Oral Health Interest Group.

